# Plasmablastic lymphoma as a manifestation of the human immunodeficiency virus: Case report

**DOI:** 10.4317/jced.56482

**Published:** 2020-04-01

**Authors:** Gonzalo Vera, Pablo Cordova, Gerson Sepulveda, Tatiana Benavides, Felipe Astorga, Wilfredo Gonzalez

**Affiliations:** 1 Residente de Cirugía maxilofacial, Universidad de Valparaíso, Valparaíso, Chile; 2 Cirujano Maxilofacial, Universidad de Valparaíso, Valparaíso, Chile; 3 Hospital El Carmen Dr. Luis Valentin Ferrada, Maipú, Chile; 4 Centro de Investigación en Ciencias Odontológicas y Médicas, Facultad de Odontología, universidad de Valparaíso, Valparaíso, Chile

## Abstract

Plasmablastic lymphoma is a rare subtype of non-Hodgkin’s lymphoma, which generally presents an aggressive clinical course and low survival rates. It is strongly associated with HIV infection and the most common site of involvement of the territory of the head and neck is Waldeyer’s lymphatic ring, followed by the gastrointestinal tract, lymph nodes and skin. The morphological characteristics of PBL in the oral cavity / jaw in the context of HIV infection show diffuse sheets of large immunoblastic cells with abundant cytoplasm, vesicular chromatin and prominent nucleus, a small located in the center with plasma cells differentiation. The main goal of this article is to review the literature of the plasmablastic lymphoma and report a case.

** Key words:**Plasmablastic lymphoma, PBL, HIV, AIDS, Non Hodgkin Lynphoma.

## Introduction

Plasmablastic lymphoma (PBL) is a rare subtype of non-Hodgkin’s lymphoma (NHL) (1), which generally presents an aggressive clinical course and low survival rates (2). The etiology of PBL is not clear, but the importance of the Epstein-Barr virus (EBV) was frequently speculated, since it was detected in 78% of the cases (2). It is well known that the nature of this malignancy, that is, the rapid appearance of the disease, the aggressive invasion in extranodal sites and the frequent repetition, even after remission, make its prognosis extremely poor (1). It was described for first time in 1997, for Stein and coworkers, who described a series of aggressive non-Hodgkin’s lymphomas (NHLs) arising in the oral cavity of human immunodeficiency virus (HIV)- positive patients (4). In 2008, the World Health Organization (WHO) accepted PBL as a special disease entity and classified it as an uncommon mature B-cell lymphoma, occurring most frequently as a mass in the oral cavity in an HIV-positive patient, but exceptions do exist (5).

PBL is strongly associated with HIV infection and other causes of immunodeficiency, including organ transplantation and advanced age. There is a percentage of male predominance (4:1), with approximately 70% and 80% of cases occurring in men, with a median age at diagnosis of approximately 50 years. However, patients with HIV infection have an earlier onset, with an average age at presentation of 38 years (3,6)

The most common site of involvement of the territory of the head and neck is Waldeyer’s lymphatic ring, followed by the gastrointestinal tract, lymph nodes and skin (1,6).

In the oral cavity, the gum was the most affected site, followed by the palate, which usually manifests as a soft-tissue lesion, which is useful for differentiating PBL from multiple plasmacytomas / myelomas that generally affect the bones. The majority of PBL present as asymptomatic swellings, frequency associated with ulcerations and hemorrhages. The most common symptoms in most cases, suggesting a more local involvement of the disease in most cases, which is consistent with the large number of cases such as Ann Arbor stage I, or currently IE in the Lugano classification; although stage IV diseases were also frequent (2).

The morphological characteristics of PBL in the oral cavity / jaw in the context of HIV infection show diffuse sheets of large immunoblastic cells with abundant cytoplasm, vesicular chromatin and prominent nucleus, a small located in the center. Frequent mitotic Figures, apoptotic bodies and blended tingible body macrophages (a “starry sky” appearance) are also characteristic of PBL, regardless of site. Confluent areas of necrosis are also occasionally present (3).

The characteristic immunophenotype seen in PBL includes the expression of plasmacytic differentiation markers including CD138, CD38, IRF4 / MUM1 (1,3), the little to no expression of leukocyte common antigen CD45 or the B – Cell Markers CD20, CD 79a, and PAX5 (2,7,8).

The most common cytogenetic abnormality observed in PBL is the reorganization of the MYC gene in 8q24, and the immunoglobulin genes act as the most frequent translocation partners (3).

A treatment difference for most cases of DLBCL (Diffuse large B-cell lymphoma), chemotherapy with cyclophosphamide, doxorubicin, vincristine, and prednisone (3,9) is generally considered an inadequate therapy for PBL (3). Instead, more intensive parameters such as hyperfractionated cyclophosphamide, vincristine, doxorubicin, and dexamethasone alternating with methotrexate and cytarabine are typically used. Because PBL shows a plasma differentiation criterion, agents that use plasma cell myeloma treatment (eg, Bortezomib and lenalidomide) have also been used to treat PBL with some success (3). Also other authors, recommend radiotherapy, with or without surgical excision, or the combination of chemotherapy and radiotherapy (9).

## Case Report

29-year-old man, heavy smoker, HIV + diagnosed 10 years ago, at that time without antiretroviral therapy. He went to the Department of Medicine and Oral and Maxillofacial Surgery of the Hospital El Carmen de Maipú, derived from periodontics due to presenting a tumoration in the vestibular gingiva between teeth 1.2 and 1.3 of 2.5 cm in diameter, of 2 weeks of evolution.

**Figure 1:**
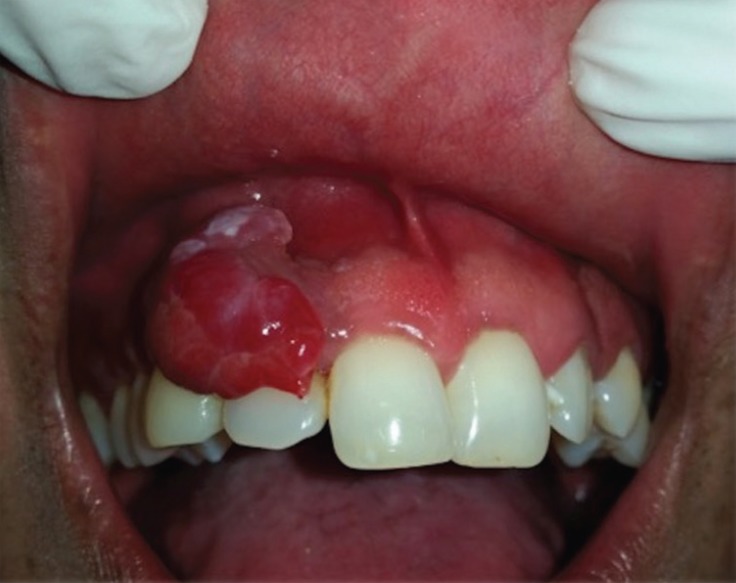
Intraoral aspect of plasmablastic lymphoma. It shows an increase in violaceous volume, ulcerated on the surface, with a partially pedicled base.

In the anamnesis, the patient referred to the minimum friction, without pain. No feeling of paresthesia. An intraoral physical examination showed an increase in violaceous volume, ulcerated on the surface, with a partially pedicled base, asymptomatic, with the presence of active bleeding. Absence of other lesions in the oral cavity (Fig. 1). In the orthopantomography, no osteolytic or radiopaque lesions were observed. No peripheral cervical adenopathies were detected on palpation.

We created a list with different possible diagnosis of the lesion, which included: Reactive Lesion (Pyogenic Granuloma, peripheral giant cell granuloma, peripheral ossifying fibroma), Benign Neoplasm (Schwannoma), Kaposi Sarcoma, Angiosarcoma, lymphoma, squamous cell carcinoma, among others.

Laboratory tests showed a clear deficiency of CD4 cells (164 cells / mm3) and a high viral load (36,000 copies / ml). The hemogram and coagulation test was normal, ESR (erythrocyte sedimentation rate) presented levels up to normal (27 mm/hr), which associates with the condition of the patient and the lesion.

**Figure 2:**
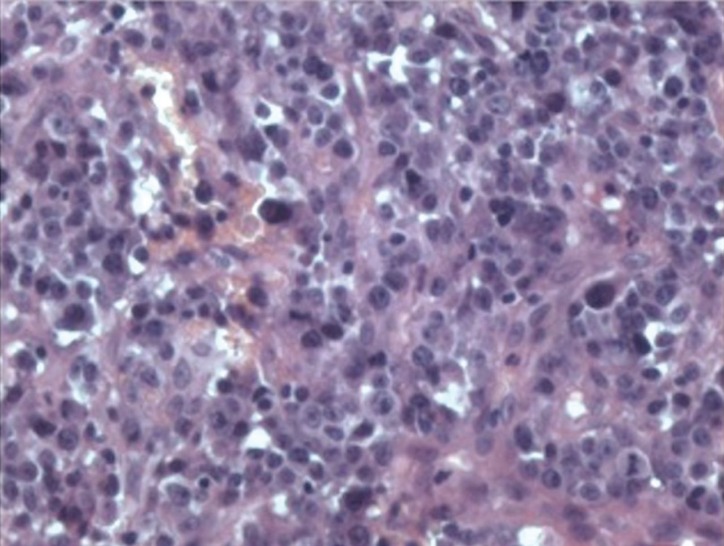
Histopathological findings. It shows an oral mucosa with a lymphoplasmacytic proliferation with diffuse disposition.

We performed an excisional biopsy with histopathological evaluation. The histopathological examination revealed an oral mucosa with a lymphoplasmacytic proliferation with diffuse disposition (Fig. 2). The examination showed plasma cells with medium size and abundant mitosis. Immunohistochemistry showed expression of CD3, CD138 and KI-67 in all cells, CD30 and CD79A in almost all cells and CD45 in a group of cells. The sample was positive for EBV. CD20 was not expressed (Table 1). The sample was positive to MUM-1 and EMA, and negative to CD56. The diagnosis was Plasmablastic Lymphoma, IE in the Lugano classification.

**Table 1:**
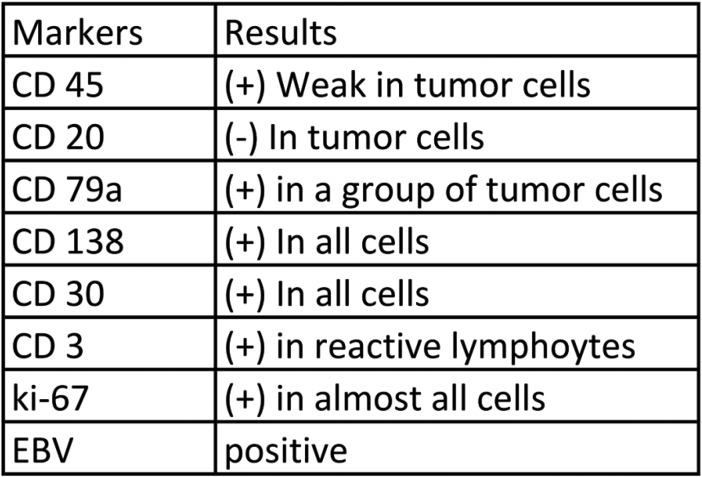
Immunohistochemistry markers.

The patient was derived to San Borja Ariaran Clinical Hospital for treatment of plasmablastic NHL and to restart therapy for HIV. The hematologist realized a marrow puncture in which they observed a bone marrow with PBL (around 20%)

The follow-up is described below.

• Cycles of chemotherapy EPOCH (Etoposide, prednisone, Viscristine Sulfate (Oncovin) and Cyclophosphamide) and ART (antiretroviral therapy) with Kivexa, Raltegravir, Norvir and Dapsone.

• 2nd Cycle Chemotherapy EPOCH with neutropenia, low-grade fever and pseudomembranous candidiasis.

The patient came to control with another lesion in the interproximal area of teeth 1.5 and 1.6 that we decided to observe according to the evolution of chemotherapy.

Four marrow punctures were performed for control:

• Two months after initiating treatment Plasmablastic LNH (20%)

• Four months after initiating treatment Plasmablastic LNH (10%)

• Six months after initiating treatment Plasmablastic LNH (5%)

• After 9 months initiated bone marrow treatment with plasmocytic infiltrate reactive aspect.

• 6 cycles of chemotherapy and remains in control by CD4 count at 120 cels / mm3

**Figure 3:**
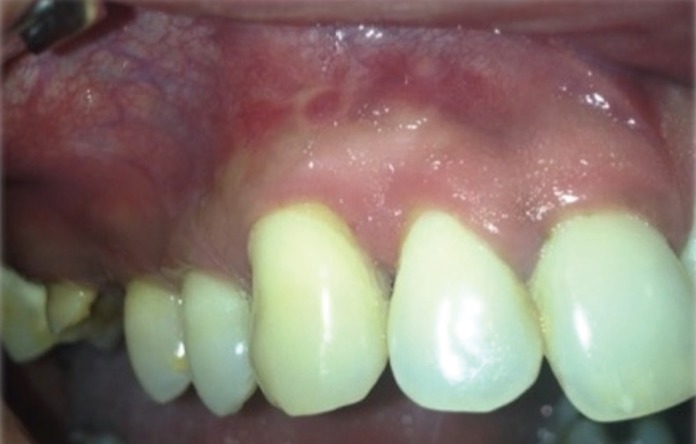
Control after the chemotherapy. The mucosa is normal.

The patient was referred for inspection of the oral cavity at the end of the chemotherapy, where no new lesions were observed, and with the Hematology pass the extraction of tooth 1.6 was programmed with a biopsy of the gingival lesion (Fig. 3). The biopsy of the lesion describe a Hyperplasia of the epitelium with fibrosis and inflammatory focus in the corium, without malignant cells. The extraction of the tooth 1.6 was scheduled because the tooth was with a Crown destruction due to dental caries with no possible rehab treatment.

## Discussion

Patients with HIV infection have a high risk of developing PBL. These lymphomas are characterized by a rapid progression, frequent extranodal initial manifestation and a precarious outcome. Antiretroviral therapy has shown a drastic reduction in the morbidity and mortality of the patient with this neoplasm. (10)

Histopathological findings in this type of lymphoma usually show a diffuse proliferation of predominantly large lymphoid cells with plasmablastic, immunoblastic or plasmocytic morphology. The number of more mature plasma cells varies from minimal to moderate in the various cases. The neoplastic cells have round nuclei, vesicular chromatin, smooth nuclear contours, prominent central / multiple nucleoli, nuclei located eccentrically variable and abundant cytoplasm (11), also associated with the expression of antigens CD38, CD138 and MUM1 (11), but not the markers expressed by mature B cells, such as CD45, CD20 and PAX5 (12). Finally, EBV was found (2).

We made a differential diagnosis with large diffuse immunoblastic B cell lymphoma, ALK diffuse positive B-cell lymphoma, primary effusion lymphoma, anaplastic plasmacytoma (plasmablastic) and germinotropic lymphoproliferative disorder associated with Kaposi’s sarcoma-associated herpesvirus (KSHV), but the previous diagnosis of HIV, the laboratory findings, the expression of lymphoid cells with CD138, CD79a, weak expression of CD45, no expression of CD20 and presence in the oral cavity, guide for the diagnosis of plasmablastic lymphoma (10,13,14).

In HIV patients, a periodical oral and cervical lymph node examination is necessary, as it can provide important information about the course of the disease.

The detection of malignant neoplasms should be early in order to provide the patient with a treatment with better prognosis in the short, medium and long term, as well as avoid possible recurrences.

The criteria of malignancy and prognosis define the treatment strategy with radiotherapy, associated or not with different chemotherapy protocols (2).

The prognosis is generally poor with a high mortality at a median of 6–7 months (6). The 5-year survival rate does not exceed 33.5%, and the presence of EBV, symptoms and the use of chemotherapy can help this poor prognosis (2).

Finally, PBL can be the first clinical manifestation of HIV infection, for this reason we must be vigilant to make an accurate and early clinical diagnosis, being able to differentiate a potentially malignant lesion from other types of oral pathology (15).
